# Origin and Development of Moral Sense: A Systematic Review

**DOI:** 10.3389/fpsyg.2022.887537

**Published:** 2022-05-09

**Authors:** Pierpaolo Limone, Giusi Antonia Toto

**Affiliations:** Learning Science Hub, Humanities Department, University of Foggia, Foggia, Italy

**Keywords:** socio-moral development, prosociality, moral judgment, affective and environmental factors, infancy

## Abstract

The literature suggests that the moral sense is based on innate abilities. In fact, it has been shown that children show the capacity for moral discernment, emotions and prosocial motivations from an early age. However, the moral sense is a complex construct of an evolutionary and social nature that evolves under the influence of interpersonal relationships. The emergence and development of moral sense is a challenge that has prompted many research studies with the aim of achieving a clear comprehension of moral development. However, success has been scarce, and studies relevant to this subject are limited. Thus, a systematic review of studies relevant to this topic was conducted to clearly establish how moral sense emerges and develops. An Ovid search was conducted to retrieve relevant items for this systematic review. The databases that were electronically visited are Cross-reference, Google Scholar and PubMed. Strict inclusion and exclusion criteria were imposed on the retrieved items to retain only relevant resources. Twenty-six studies were found valid for inclusion in this systematic review. The results of these studies were presented differently: In order to effectively analyze the selected papers and bring out the results more clearly, a categorization of the approaches adopted in the studies was carried out. The approaches identified were: “Natural Moral Sense,” “Social Relationships and Moral Development,” and “Environmental Factors and Moral Development.” The evidence that emerged from the analysis of the papers was collected to produce a general basic model that explains moral development while also serving as a link between the various studies. First, moral sense is found to be innate in humans; individuals can naturally respond morally to various dilemmas. As seen among children and young infants, moral sense naturally exists. Second, it can be socially nurtured through social interactions and exposure to various environmental factors. Various research studies were reviewed in this systematic review to obtain a consensus on how moral sense emerges and develops. From the systematic review, the moral sense is found to be innate. However, moral development is fostered by social interactions and environmental factors.

## The Origin and Development of Moral Sense: A Systematic Review

As many researchers have come to realise through their studies, it is difficult to try to comprehend morality clearly. A conclusion on whether morality is natural or socially developmental has still not been obtained, given the wide range of constraints, such as a limited number of related findings and compromised results (Globokar, 2018). Researchers have found this topic rather convoluted, with a number of them failing to categorise moral sense as either a natural either natural, therefore innate, or a socially-progressive process, i.e., emerging in response to interpersonal relationships. It is, therefore, possible to distinguish the two main lines of research that investigate the nature of the moral sense: innatism and socio-constructivism. The first considers the innate moral sense, which is present from birth; the second theorizes that this function is the result of a social construction influenced by the interpretation of the subject of lived experiences. It should be noted that the two positions are completely opposite: considering the moral sense as an innate function does not mean rejecting the idea that morality cannot change and evolve during growth (Singer, 2019). Innatism, therefore, asserts that a natural basis of morality is associated with the subsequent processes of accommodation. On the other hand, socio-constructivism believes that moral sense is the result of learning determined by social relations. However, it is also the result of the evolutionary process of the human species.

Socio-constructivism states that Moral development is a process by which human moral sense progressively identifies and distinguishes between the proper and improper, the right and wrong (Turiel, 2015). The moral sense of individuals is considered to be developmental. It progresses over time, distinguishing simple to complex definitions. Evolutionary studies suggest that human morality derives from group selection: during its evolution, the human species encountered socio-ecological conditions that made group selection the dominant evolutionary force. In fact, natural selection has favoured the development of cognitive abilities, emotions and motivations essential for cooperation within groups. Morality, therefore, arises from the human need to preserve the bond with other people and to take care of them to survive (Fimiani et al., 2018). From an innate and evolutionarily founded design, morality is subsequently shaped by social experiences and interpersonal interactions. The human moral sense advances from defining the finite right and wrong to more intricate ways of differentiating between the two. According to Dorough (2011), the human moral sense is never static, and continuous advancements characterise it. Cherry (2021) affirms this, stating that moral development is a life-long process. Triggered by social interactions and a wide range of environmental factors, people’s moral senses are constantly developing.

In support of innatism it is a fact that when infants are born, they know almost nothing about the social environment. As time goes by, without parents necessarily teaching them, infants develop some forms of judgement on what is viewed to be morally upright. At the age of one, young children are seen to support themselves with chairs and erect materials to stand. During this period, most young children also start to walk by themselves without interventions (Tomasello, 2018). These behaviours show that even before the age of three, children have a tendency to prosociality which subsequently turns into morality due to the learning of normative standards. They are seen to respond to their needs by crying or smiling socially. Additionally, grown-up children are observed to help their parents and guardians accomplish their daily tasks. Thus, they seem to have internal moral judgements that motivate them to make such social responses to the environment and people surrounding them. This observation establishes the innateness of moral development (Laible et al., 2019). According to Lehalle (2020), explaining morality as either natural or socialised is challenging. Despite this constraint, researchers have identified that morality emerges in a progressive system (Turiel, 2018). The moral senses of children are continually shaped by the relationships built around them. With these relationships, children are able to socially interact with individuals who will ultimately boost their moral progression. For example, they can improve their moral senses by interacting with caregivers, who are considered to be the best builders of children’s morality.

The findings of Yusuf et al. (2018) are supported by Lawford et al. (2021), who argues that moral judgement is a very organised subject that clearly distinguishes different ages of children. According to Lawford et al. (2021), older children demonstrate a higher moral understanding of harm than younger ones. This indicates that as people grow, so do their moral judgements. Through interacting with social aspects, people inculcate moral judgement skills and develop them with further social interactions.

For many years, researchers have closely studied this topic to establish a clear understanding of human morality. In fact, it has always been difficult to make the right decisions on what is proper or improper. Moral judgements are part of our daily lives. Every individual is always tasked to judge various social aspects of life correctly. According to Willer and Simpson (2017), moral judgements are sometimes considered to be against the social codes of contact. From these considerations, a new path opens up with respect to antisocial behaviours, which, however, is not the subject of discussion in this review but is noteworthy for the completeness of the analysis: some of the individuals in society at times make judgements that are not in agreement with social norms. Politicians, for example, sometimes make moral judgements that are contrary to the social settings. Thus, it can be said that moral judgements are sometimes antisocial (Kim, 2018; Noveni et al., 2021).

The minds of humans are considered to be biologically prepared as children from an early age show moral evaluations and prosocial behaviours. However, during development, they are influenced by the socio-cultural context. When exposed to different aspects such as social interactions and environmental factors, moral senses develop. Hamlin (2013a) observes that although some aspects of moral sense may have evolved to promote cooperation for survival, an innate moral core exists in preverbal infants and children, which structures sophisticated and flexible moral behaviours and assessments, particularly the ability to identify and evaluate others based on their prosocial or antisocial acts within the first year of their life (Hamlin, 2014). Morality is also evident in the animal species; for example, in chimpanzee groups, the members celebrate only after the power struggles have been resolved: the group desires peace and prefers harmony rather than cooperation and sustenance. Humans experience the same feeling even before experiencing and learning from their fellow men (Haidt and Joseph, 2007).

Dawkins et al. (2019b) also confirm the innate nature of morality: at least four socio-moral principles prematurely guide children’s reasoning and expectations, such as fairness, avoidance of harm, support for the group and respect for authority.

In support of this perspective, CK-12 Foundation (2016) states that young children from the age of one to three learn many things through exposure. For instance, they are able to portray various responses that they learn from their superiors. Additionally, a significant change occurs when young children reach adolescence. Adolescents show much interest in their friends and peers. At this stage, these individuals are still learning how to manage their emotions (Lerner et al., 2015). Their peers’ interactions greatly influence them CK-12 Foundation, 2016. Adolescents begin to form intimate relationships during this phase. Thus, through interactions with social peers, people learn and develop their moral judgement. This proves that moral development is socially progressive rather than naturally developmental: Interpersonal relationships and the socio-cultural context are decisive as they influence and modify the moral sense. Therefore, it is not possible to consider the function regulated only by the natural development of the human mind.

From the above emerges the constructivist perspective. According to this, each person gives meaning to their own experience and takes actions in relationships based on a set of personal premises and beliefs, which derive from their specific position in the interactive situation, from their previous experiences with relationships. There is no single, universally applicable set of moral requirements; rather, moral requirements are diverse and apply to different people according to their own experiences, emotions and motivations, and socio-cultural context (Brink, 2003). According to Sommerville and Ziv (2018), an intuitive sense of fairness emerges within the first 2 years of life and encompasses many aspects of mature moral responses. Specifically, the authors demonstrated that in children, an intuitive sense of fairness emerges as a result of experience and is derived from their interactions, which allow them to observe and participate in social exchanges, assuming the role of both agents and recipients of fair and unjust behaviour.

Therefore, this systematic review seeks to answer the question: How does moral sense emerge and develop? It is also necessary to establish a clear consensus on whether moral development is wholly natural or socialised. In this particular review, the study aims to establish all possible contributing factors of the emergence and development of moral sense in humans.

## Methodology

This particular systematic review is aimed at establishing a clear understanding of the topic: emergence and development of moral sense. It also focuses on ascertaining whether moral development is innate or socially developmental.

## Protocol Development

A proper protocol must be consistently implemented to establish a stable background for responding to the review question articulated in any systematic review. For this review, multiple protocols were shaped to fully answer the questions under consideration. All the required review scopes were considerably added to this protocol. They comprised the search strategy, inclusion criteria, screening methods, data analysis and synthesis. The Preferred Reporting Items for Systematic Review and Meta-analysis (PRISMA) model was utilised in this systematic review.

## Eligibility: Inclusion and Exclusion Criteria

### Reporting Language

A crucial eligibility criterion for this systematic review is the reporting language. All the resources necessary for this review were supposed to have been reported in English. All articles and other resources reported in other languages were excluded from this systematic review irrespective of their usefulness.

### Type of Study

The studies to be included in this systematic review had to present information contributing to socio-moral development. Research studies using quantitative methods compatible with theoretical approaches were considered eligible for inclusion in the systematic review. Any other studies contrary to the above information were automatically removed from the inclusion list.

### Outcome Measures

All the studies that reported positive moral development as an outcome measure were added to the inclusion list for the systematic review. Studies and resources that were not aimed at reporting on this particular outcome measure were automatically excluded from this systematic review.

## Search Strategy

The retrieval of valid results from databases is the primary goal of every search strategy. A researcher can obtain accurate results only if an organised structure is utilised in the search process. Numerous strategies are available for use during the search process. These search strategies include Ovid search, the use of Boolean logic, citation search and the subject headings search approach. Despite the wide range of methods, inaccurate results occassionally curtail the efforts of researchers. The Boolean logic allows the appropriate combination of operators (and, or, not, adj, near, then, etc.) for the database query syntax (Catania, 2004). With a basic search on Ovid, it is possible to access full-text open access journals from different databases by adopting specific operators. The citation analysis is the cornerstone of the research discipline known as bibliometry, which operates through the following indicators: Impact Factor, H-index and its variants, Eigenfactor, Scimago Journal Rank, and SNIP (Cassella and Bozzarelli, 2011). Subject headings are an alternative to text word scanning that can be used for searches within the journal record. Subject headings constitute a thesaurus that involves a more precise search but at a lower sensitivity cost than text words (Jenuwine and Floyd, 2004).

The search strategy utilised for this systematic review is an Ovid search. The search process included visiting a number of databases, as far as the retrieval of accurate search results was concerned. Among the databases, Cross-reference, Google Scholar and PubMed were considered relevant for finding studies that accurately fit the scope of this systematic review. During the process of searching, the hand search technique was used. Only the studies published in the English language were eligible for inclusion. In the search process, prosociality, moral judgement, socio-moral development, affective and environmental factors and infancy were considered keywords crucial for the retrieval of valid search results.

## Study Selection

The selection of studies to be included in the systematic review is very complicated; valuable resources need to be identified for the review to be valid. This is achieved by utilising relevant selection techniques that will ultimately return relevant studies. With a massive number of search outcomes, a screening process needs to be conducted to identify both the valid and invalid articles for the systematic review. In this systematic review, title and abstract screening were conducted on the search outcomes to sieve out the relevant studies – this involved screening the articles’ titles and abstracts based on the inclusion criteria. For the purpose of the exclusion, full-text analysis was conducted.

Two reviewers critically and independently reviewed the eligible studies, especially the abstracts and titles. The main aim of the two independent reviews was to come up with a reasonable consensus on whether the studies could be included in the systematic review. The reviewers tabled the different resources to initiate a discussion on which articles were eligible for inclusion. A discussion between two reviewers is a two-way process, which can result in both agreements and disagreements. In case of the latter, a third reviewer should be selected as a backup, who would intervene in the discussion when a consensus could not be reached. In this systematic review, a third party played a significant role in settling disagreements that arose between two independent reviewers. Ultimately, a valid consensus was achieved, thanks to the third reviewer.

## Data Extraction

A suitable data collection process is a necessity of every systematic review – all fields required need to be included in the data collection. The MS Access database was used to collect data in this systematic review. The fields captured by the data collection database were study design, participants, inclusion and exclusion criteria, methods and results. The independent reviewers were further tasked with collecting and recording the required data based on the fields highlighted above. The third reviewer revised the data to confirm its relevance. In cases of missing information or confusing data, the primary authors of the studies were contacted to harmonise such situations. Qualified articles were forwarded for systematic review.

## Risk-Of-Bias Assessment

The two independent reviewers participated in assessing the risk of bias of the studies selected. To conduct the process in the most effective and relevant way possible, the Cochrane risk-of-bias tool (CRBT) was used. The CRBT instructions contain systematic strategies to reduce bias as the conclusion can become unreliable if the data are incorrect. Thus, an assessment of the internal validity of the included studies is made to reduce the likelihood of bias. The tool is based on seven bias domains: sequence generation and allocation concealment (both within the domain of selection bias or allocation bias), blinding of participants and personnel (performance bias), blinding of outcome assessors (detection bias), incomplete outcome data (attrition bias), selective reporting (reporting bias) and an auxiliary domain: “other bias.” For each bias domain, the tool urges users to assign a “high,” “low,” or “unclear” risk judgement of bias and to document the basis of their judgements (Jørgensen et al., 2016).

The use of this criteria list is highly recommended to reviewers during the risk-of-bias assessment process; therefore, this review utilised it. The CRBT comprises items that are useful when assessing the validity of the selected articles. The search resources in agreement with the CRBT demands were awarded positive scores, whereas studies contrary to the CRBT were given negative scores. All the records were discussed by the independent reviewers during the consensus meeting. Studies meeting the recommended items were regarded to have a low risk of bias, while articles not meeting the recommended items were observed to have a high risk of bias. A study was considered to be eligible to be included in the systematic review only if it had a low risk of bias. On the other hand, studies with a high risk of bias were considered ineligible and were excluded from the systematic review. To reach an agreement, the disagreements of the reviewers were resolved by a third reviewer.

## Data Analysis and Results Synthesis

A systematic review was performed based on the Cochrane requirements. Search outcomes were compared for the systematic review; articles were pooled and analysed through a 95% confidence interval (CI). The search articles were analysed based on the three inclusion criteria: type of study, outcome measures and reporting language. This only concerned homogeneous studies. To check for the heterogeneity of articles, a *Q* test was performed.

## Publication Bias

A systematic review, by all means possible, always has to be valid. However, the validity of a review is sabotaged by the presence of publication bias. Therefore, publication bias needs to be fully explored in a systematic review for it to be justifiable. In this case, the construction of funnel plots aided in determining the possible publication bias of the included studies.

## Results

### Search Outcomes

**FIGURE 1:**
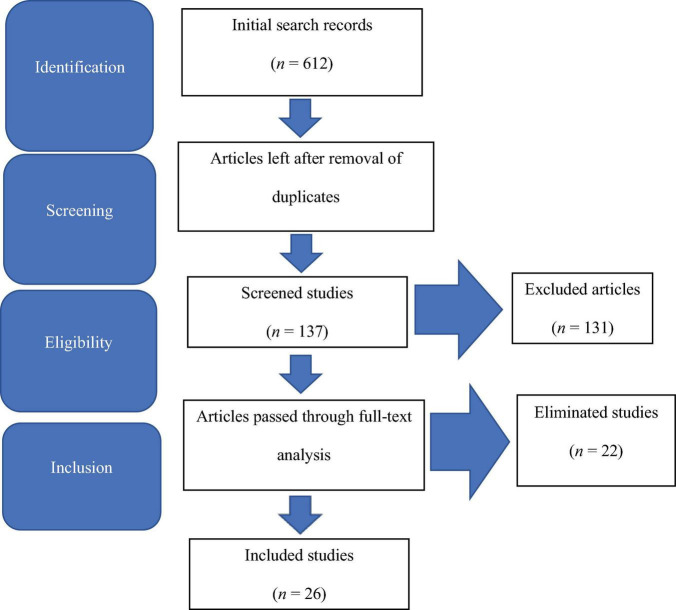
Preferred Reporting Items for Systematic Review and Meta-analysis (PRISMA) model.

A total of 602 studies were retrieved electronically from the search databases, Cross-reference, Google Scholar and PubMed. In the preliminary screening, all 602 articles were used, and then, 433 articles were removed as duplicates. Furthermore, 131 studies were eliminated based on invalid abstracts and titles. Till this stage, 48 studies survived the elimination process. All 48 articles were tested on the eligibility criteria, and 22 articles were eliminated after a complete text analysis was done. The remaining 26 studies were consistent with the inclusion demands and were, therefore, considered for the systematic review. Figure 1 presents the PRISMA model utilised for the search strategy in this systematic review. Table 1 below shows the main information of the papers.

**TABLE 1:** Overview of reviewed studies.

N.	Author and year of publication	Type of article	Theme	Results
1				
hboxcitealpBR24	Experimental study	The relationship between preference and social causality	The results demonstrated that children’s assessments are triggered by their perception of social causality: only in the social condition did children expect a spectator to help an intermediary agent	
2	Korotaeva and Chugaeva, 2019	Review	Social and moral education and development of preschool children	The social and moral representations of children are tools for understanding their actions and their relationships with adults
3	Franchin et al., 2019	Experimental study	Beginning of the activation of the moral domain	The results suggest that within 30 months, children began to associate good with some socio-moral characteristics, such as a helping disposition, but not with equity in distributive actions
4	Hamlin, 2013a,b	Review	Moral evaluations in infants	The ability to distinguish between intentions and outcomes in morally relevant events is present at the age of 8 months
5	Hamlin, 2013a,b	Review	Behaviour and moral evaluations	Human morality is a fundamental and innate aspect of human nature, as it allows for cooperation
6	Geraci and Franchin, 2021	Experimental study	Children’s reaction to agents in need and not in need of help	When agents in need are morphologically similar to agents in need, children watched the event longer, which showed that agents in need help agents in need
7	Surian et al., 2018	Experimental study	Children’s reaction to a character who harms another agent	Children who saw the helper perform an unequal distribution looked longer than those who saw the helper perform an equal distribution, while children who saw the hinderer perform an unequal distribution looked the same way as those who saw the hinderer perform an equal distribution
9	Lavoie et al., 2021	Review	Methods for measuring moral development	The review revealed the presence of an innate moral sense in children under 2 years of age
10	Holvoet et al., 2016	Review	Preference for prosocial behaviour	Innate preference for prosocial behaviours
11	Ting and Baillargeon, 2021	Experimental study	How young children value other characters on a moral level	Children from an early age develop moral assessments that regulate their behaviour
12	Denton and Krebs, 2017	Review	Moral decision-making process	The ways in which brain mechanisms have evolved and developed throughout life provide a basis for explaining why people possess the ability to engage in moral decision-making
13	Dahl and Baxley, 2016	Chapter in a book	Morality development in early childhood	The main stages in the development of morality are retraced in this work
14	Hilton and Kuhlmeier, 2019	Review	Moral evaluation	At the beginning of development, humans evaluate others by considering the outcome of an action in relation to the intention behind it
15	Chiasson et al., 2017	Experimental study	Change in socio-moral reasoning during evolutionary development	The study found a linear increase in moral reasoning (MR) from infancy to late adolescence, with significant group differences between childhood (6–8 years) and pre-adolescence (9–11 years)
16	Baker et al., 2022	Article	Differences in the morality of individuals with conduct disorder and healthy individuals	The factors of “intelligence” and “maternal support” exert a decisive influence on the level of socio-moral development
17	Hammond et al., 2021	Clinical trial	Educational programme for abused teenagers	After the application of the educational intervention programme on self-control and moral development, the experimental group showed fewer learning difficulties and less tendency to aggression
18	Goffin et al., 2020	Article	Mind-mindedness of the mother and children	It demonstrates the positive association between the mind-mindedness of the mother and that of her children and the theory of mind of the children
19	Dahl, 2019	Review	Development of the tendency to help and harm	Children’s orientations towards helping and harming others gradually develop through daily social interactions in the early years
20	Emler, 2021	Chapter in a book	Moral development in children	It outlines children’s socio-moral development from the point of view of social representations
21	Bian et al., 2018	Experimental study	Fairness in cases of limited resources	Infants expect ingroup support to override fairness when resources are limited
22	Dawkins et al., 2019a,b	Experimental study	Fairness in children	Sensitivity to fairness is part of the basic cognitive structures of a human being
23	Tan et al., 2021	Article	Moral functioning and preschool children	Empathic worry and inhibitory control have emerged as important predictors of the moral functioning of preschool children
24	Hamlin and Van De Vondervoort, 2018	Article	Infants and children’s preferences for prosociality	Preverbal children evaluate third parties based on their morally relevant acts, considering other prosocials positively and negatively evaluating other antisocials
25	Govrin, 2014	Review	Moral function in infants and young children	Moral function is innate, but in the first years of life, it develops in response to interactions with the caregiver
26	Geraci et al., 2021	Experimental study	Moral evaluations of preschool children	Children prefer the agent who consoles the victim over the one who presents indifference

## Analysis of Risk of Bias

Risk-of-bias analysis was conducted in this systematic review to discover the methodological quality of the articles included. All the included resources were found to be of adequate methodological quality; they were all regarded as qualified for systematic review. The sufficient methodological quality of the articles was accepted by the two independent reviewers as accurate and was then confirmed by the third reviewer.

## Characteristics of the Studies

All the studies included in this systematic review provided sufficient information contributing to socio-moral development. Additionally, studies exploring methods that are in accordance with the different theoretical approaches are selectively included in this systematic review. All studies included are reported in the English language.

## Individual Results of the Studies

The studies included in this systematic review explored the emergence and development of moral sense in humans. The theoretical approaches of reference of the studies differ from each other as well as the type of paper: the theory of innatism or socio-constructivism guide researches and interpretations of the data in a different way. Moreover, it should be noted that the studies have the nature of review, experimental study, clinical trial, and chapter book. They use different methods respective to different approaches to contribute to moral development. Their conclusions are presented as follows.

### Natural Moral Sense

The research problem of whether moral sense is wholly socialised or natural has, to date, not been settled despite the numerous studies conducted on this problem. However, quite a few studies have shown that infants and young children exhibit different moral skills at a young age. One-year-old children are seen to explore their social environment by employing inherent skills. According to Carpendale and Hammond (2016), children always help their parents in their daily activities. They are seen to actively take part in fulfilling daily obligations without necessarily being instructed to do so. This shows that young children possess natural moral abilities to decide what is good and what is not. They distinguish between the virtue of helping parents and the wickedness of staying idle. Other researchers have also interpreted that morality emerges naturally in humans (Carpendale and Hammond, 2016). Moral sense is naturally present in a human being, irrespective of their age. Infants and young children possess natural morality that allows them to distinguish between the simple definitions of proper and improper.

A study to assess the moral judgements of young children was also conducted to establish how children view moral transgressions (Smetana and Ball, 2018). In this study, young children aged between 4 and 9 years were tested to determine their response to physical harm, resource transgressions and psychological harm to their enemies and friends. Among the children, transgressions against their enemies were more accepted than those against their close friends. Moreover, the transgressions were least intended when targeted at close friends compared to enemies. Smetana and Ball (2018) confirmed that children considered moral transgressions as improper and wrong. This establishes that the children, across all relationships, distinguish between right and wrong or good and bad. Thus, moral sense is considered to be natural.

Smetana and Ball (2019) conducted another study to ascertain whether age is a determining factor in children developing their moral judgements concerning different types of harm. This study concentrated on children aged 4–9 years and sought to determine their moral judgements from age profiles based on different types of harm. Ultimately, the authors found that children of different ages portrayed significant differences in moral understandings regarding other forms of harm (Smetana and Ball, 2019).

Young children, aged four, depicted little moral understanding of different types of harm. On the other hand, grown-up children, aged nine, had a greater moral understanding of these. In general terms, Smetana and Ball (2019) confirm that children’s moral judgement is organising according to their ages. Young children made weak moral judgements, whereas older children made strong moral judgements. This shows that children’s moral judgements develop naturally with respect to their ages. 160 children aged 4–9 were observed to evaluate the emotions, judgements, intentions and justifications they manifested with respect to physical and psychological damage and transgressions aimed at acquaintances, friends, disliked peers and bullies. Transgressions against bullies were found to be more acceptable among the children than those against friends and disliked peers and were deemed to be less deserving of punishment. In addition, the children viewed moral transgressions as wrong based on the principles of well-being and fairness.

Geraci and Simion (2021) investigated whether the perception of the social-causal relationship triggers both children’s evaluation processes and expectations relating to the social preferences of third parties. To this end, three experiments were implemented that reproduced a social causality, a physical causality and a choice task to assess children’s preferences.

Research results (Geraci and Simion, 2021) have demonstrated that children’s evaluations are triggered by their perception of social causality: Only in a social condition did children expect a spectator to help an intermediary agent. Explain the children’s actions and their relationships with each other and with adults, and then, evaluate these actions. Korotaeva and Chugaeva (2019) focus on the study of the moral representations of preschool children. This experiment was conducted among preschool children in one of the groups of the Ekaterinburg kindergarten. The theoretical basis of reference was that social behaviour is characterized by duality: on the one hand, a person’s actions are determined by social standards and norms; on the other hand, the actions are controlled by the person and his/her ability to choose and take responsibility for that choice. During childhood, a child experiences social relationships, learns to solve problems and assimilates moral standards through personal perception. The experimental study demonstrates the importance of developing methodical recommendations for interactions between teachers and children, or between parents and children, to mediate effective social and moral education that takes into account the children’s perspectives on moral standards and rules of conduct. Thus, the authors highlight the importance of adults in the development of moral sensibility by respecting the personal ideas of the children. Franchin et al. (2019) suggest that children within 30 months begin to associate goodness with socio-moral characteristics such as a helping disposition but do not do so regarding equity in distributive actions. The ability to distinguish between intentions and outcomes in morally relevant events is present at the age of 8 months (Hamlin, 2013a). The “moral mind” of the young child is fundamentally different from that of older children and adults. However, as early as 8 months, children incorporate, and even prioritize, intentions in their social assessments. Instead, 5-month-olds appear to be able to distinguish only the characters who intend the outcomes they cause.

Hamlin (2013b) also argues that human morality is a fundamental and innate aspect of human nature, as it supports cooperation. This mental function has developed with the evolution of the human being who needed to aggregate to survive. Hamlin demonstrates that infants naturally engage in surprisingly sophisticated and flexible behaviour and moral assessments.

A section of these studies concerns how individuals can naturally respond morally to various dilemmas: Geraci and Franchin (2021) investigated whether 21-month-olds expect non-needy agents to help agents in need (type of puppet) of food or shelter. The results show that when agents in need are morphologically similar to agents in need, children watched the event longer, which showed that agents in need help agents in need. Fourteen-month-old babies were familiarised with a character who helped or hindered another agent’s attempts to reach the top of a hill. The hinderer condition started with the same familiarization phase used in the helper condition, but in the test phase, the distributor of the strawberries was the hinderer instead. The preliminary analysis assessed the effects of the order of familiarization events (Help, Hinder, Hinder, Help vs Hinder, Help, Help, Hinder) and the identity of the helper and the hinderer (Square vs Triangle). It found that they had no main effect on looking times at the test trials, nor was there a significant interaction between such factors and the type of test event (equal vs unequal distribution). Children who saw the helper perform an unequal distribution looked longer than those who saw the helper perform an equal distribution, while the children who saw the hinderer perform an unequal distribution looked the same way as those who had seen the hinderer perform an equal distribution (Surian et al., 2018). Lavoie et al. (2021) confirm the findings of previous studies through a review centred on the methods used to measure the moral development of children under the age of two. They show that moral sense is innate, that is present from birth.

### Social Relationships and Moral Development

As mentioned earlier in this paper, people’s moral sense develops over time due to social interactions. In this context, children are considered to encounter moral development when they interact with their parents or even peers. According to various studies, the issue of the emergence of the moral sense has continued to be unclear, and whether moral development is wholly socialised or naturally acquired is a topic of debate still yet. Nonetheless, studies have found that the uniqueness of interactions significantly contributes to moral development. In a social context, moral knowledge is enhanced when a person is exposed to others’ views and ideas. An individual can grow and develop morally only if the social interactions experienced are educative. In general terms, the active discussions and negotiations a person participates in are the pillars of moral development.

Govrin (2014) produced a general model of the process that contributes to the development of moral judgement in the first year of life, whereby a universal and innate structure of the moral faculty is hypothesised, which is therefore pre-reflexive. However, the latter develops through early interactions with the caregiver. The internal representations that the child constructs in the dynamic system with the caregiver determine their judgements about what is wrong and what is right. This work suggests explaining the mind in the first year of life by integrating Bowlby’s attachment theory, ethics of care and moral psychology that were previously regarded as separate domains.

Another literature review was conducted by Carpendale and Hammond (2016) to pinpoint the source of moral norms among children and infants. The authors sought to examine previous claims that proposed that infants have a natural moral understanding. While examining this information, the study aimed to establish the source of the children’s moral knowledge—whether it occurred naturally or was socially nurtured. In the review, Carpendale and Hammond (2016) categorically presented the views of the previous researchers. According to them, previous studies stated that children help their parents – young children are always seen to take part in daily activities. Moreover, they put forward that children are interested in pleasing people but are scared away by bad people. This has been interpreted as human beings possessing natural morality.

However, the aforementioned explanation from Carpendale’s review has given rise to more questions than solutions. As in the above case, the source of morality is still unclear – whether it is wholly socialised or natural.

Geraci et al. (2021) evaluated the reactions of preschool children in different situations of social interaction. They show that children preferred the character who consoled the victim over the one who did not. Thus, it emerges that social assessments are based exclusively on social interactions between prosocial/antisocial agents and recipients. Furthermore, the authors’ findings reveal an evolutionary tendency in children to reward the puppet who comforts and punish the puppet who ignores the victim.

Carpendale and Hammonds’ study Carpendale and Hammonds’ (2016) also concentrated on examining the source of this moral sense. The daily interaction between children and caregivers is seen to significantly foster children’s ability to coordinate morally with others (Cingel and Krcmar, 2020). Through social interactions between them and their superiors, children are able to equip themselves with sufficient moral skills and ultimately nurture their inbuilt moral senses. The authors conclude that children’s moral development is significantly boosted by the continuous daily interactions they have with parents and guardians. Thus, according to Carpendale and Hammond (2016), moral development is socialised, that is social interactions contribute to morality enhancement.

Mammen et al. (2019), in their research study, focused on how children develop their moral judgements through socially interacting with their parents and peers. This study sought to investigate the differences in talking about moral problems when children interact with their parents and their peers. With their parents, the interaction is seen to contribute little as far as the moral development of the children is concerned (Mammen et al., 2019). Children are depicted in Mammen’s study as lacking enough freedom to talk about their parents’ problems. Mothers and other adults do not always negotiate or discuss moral challenges with their children. This, therefore, denies children the opportunity to develop moral skills for tackling different moral issues.

Conversely, children are seen to negotiate and discuss their dilemmas with their peers freely. They believe that with their peers, who are of an equal level, moral problems can be actively discussed. This, therefore, enhances their experience and, in turn, develops their moral senses. Based on these perspectives, social interactions, especially with peers of an equal level, give individuals the best opportunity to negotiate their moral challenges. This develops them morally, hence contributing to their moral sense. Studies (Chiasson et al., 2017) have focused on changing socio-moral reasoning from infancy to adolescence, and results show that boys with conduct disorders tend to differ from their healthy counterparts in terms of their level of socio-moral maturity of judgement. The factors of “intelligence” and “maternal support” are known to exert a decisive influence on the level of socio-moral development (Baker et al., 2022). The main goal of Hammond et al. (2021) was to show the results of an educational programme on self-control and moral development taught to a sample of abused and abandoned adolescents. Another aim of this subject concerns socio-moral development from the point of view of social representations (Emler, 2021). Significant interpersonal relationships make it possible to construct representations of oneself, of the Other and of the World by mediating cognitive schemes that filter the interpretations and evaluations of one’s own and others’ actions. This process structures the moral sense in the child who develops and matures in the first years of life.

Goffin et al. (2020) experimentally demonstrated the positive association between the mind-mindedness of the mother and that of her children and the theory of mind of the children. Finally, Dahl (2019) examined research on how children’s orientations towards helping and harming others gradually develop through daily social interactions in their early years.

Concerns for the welfare of others, rights, equity, and justice serve as the foundation for structuring the moral sense for the judgement of what is right and wrong. The authors affirm that this passage from a primary to a complex moral is allowed by daily interactions. Therefore, morality is neither innate nor solely the result of caregiver relationships.

### Environmental Factors and Moral Development

The environment plays a significant role in shaping the moral sense of human beings. Behavioural expectations are made clear to children when the physical environment is set appropriately. The physical environment provides young children with expectations of behaviour. When educators are aware of the aesthetics, organization, and function of each area of the space, defiant behaviour is likely to decrease while constructive and cooperative behaviour increases. A well-designed curriculum provides the best opportunity for children to access a wide range of items and ideas that reflect their interests. This great accessibility allows young children to freely discuss their interests with each other. The environment’s design is crucial as far as moral development is concerned (Paris et al., 2021). For young children, a space of tremendous and well-designed learning is constructive and interactive. Such an environment has learning materials placed at a location readily accessible to individuals, and children can freely utilise the materials based on their interests. They can negotiate with each other over the materials they have. Additionally, ample space enables child–adult interactions. This creates an opportunity to expand interactions and discussions. The expansion of discussion and negotiation boosts the moral abilities of the people involved in the conversation.

A well-designed physical environment is always observed to be socially attractive. According to Paris et al. (2021), a learning environment designed with quality in mind sets the best background for boosting socio-emotional development. The preparation of a high-quality environment with sufficient learning items is attractive to children and adults. This attraction allows the concerned individuals to participate in activities of their interests and abilities fully. This, in turn, further develops their moral senses. An environment that enables moral development comprises ample and diversified learning space, enough supply of material, supportive engagements and displays of individuals’ work.

A socially attractive environment must possess a number of user-friendly factors, such as ample interaction space, and it should be well equipped with the necessary resources. An individual exposed to this space is free to make moral judgements based on the available resources. Notably, a person plays with the environmental resources based on their interest and ability. This contributes to moral development through enabling constructive interactions and engagements. As stated, it allows individuals to freely explore their interests and abilities by utilising the ample resources available to them. Therefore, young children are allowed to manage their relationships and themselves fully. Through this, they are able to develop morally. Environmental factors, consequently, enable moral development.

The social environment is a precursor to morality and moral development (Cowell and Decety, 2015). A study conducted by Cowell and Decety (2015) found that socio-environmental factors support the sensitivity of moral sense among toddlers and young infants. As confirmed by Cowell and Decety (2015), the minds of young children are biologically prepared. They are already in possession of moral sense. Human beings are biologically predisposed to the construction of moral judgements and evaluations, which, therefore, structure behaviours aimed at cooperation and associations between antisocial actions and punishments. The interaction between the environmental factors and these infants’ minds impacts their behaviour and social evaluation (Cowell and Decety, 2015).

This is the result of their study that looked at the neural basis and precursors of moral sensitivity in 73 subjects aged 12–24 months. The analyzes performed were of electrophysiological, eye-tracking, behavioural and socio-environmental nature. The study established the interaction of prepared minds with supportive environmental factors to be a great contributor to socio-moral development. The social and moral behaviours of the involved individuals develop when exposed to various environmental foundations. In other words, a person exposed to various supportive factors is well-positioned to behave differently from their previous behaviour; this is moral development. Considering the above perspectives, environmental factors contribute significantly to moral development. Two specific areas are evaluated in the study by Holvoet et al. (2016): (1) studies that have previously explored social assessment skills beyond a basic preference for prosocial behaviour and (2) current theories attempting to explain how and why such preferences could exist so early in childhood. Ting and Baillargeon (2021) explore how young children value other characters on a moral level and reveal how these ratings, in turn, allow children to form sophisticated expectations about the behaviour of others in new contexts. Denton and Krebs (2017) examine the psychological and neurological evidence supporting dual moral decision-making models and discuss research that has attempted to identify triggers for rational-reflexive and emotional-intuitive processes. Dahl and Baxley (2016), on the other hand, present the main theoretical perspectives on the early development of morality and its basic elements during the first 4 years of life. Finally, research on infants and young children suggests that even during early development, humans evaluate others by considering the outcome of an action in relation to the intention behind it. On this note, Hilton and Kuhlmeier (2019) review existing research on moral evaluation and propose that differences in how intentions and outcomes of behaviours are viewed may (1) support or preclude attribution of intentions to young children and (2) alter the relative relevance or predominance of any type of information.

In conclusion, the studies that can be categorized in the socio-environmental approach consider the environmental and social factors determining moral development, but they also agree on the existence of a predisposition to prosociality present from birth.

## Discussion, Limitations and Conclusion

### Discussion

This systematic review included 26 studies. Although these studies used different methodological approaches, all of them contributed to the topic under study. Neverthless, the emergence and development of moral sense is a theme that still requires further study, as studies focusing on this area are few and far between. The facts and research available claim that moral sense is naturally occurring (Garrigan et al., 2018). Infact, many researchers have found it difficult to determine the source of morality in humans – whether moral sense is wholly socialised or natural. This stand is also consistent with Kotlova (2020) findings that children view moral transgressions as being wrong. These interpretations thus converge, indicating that morality and moral judgements are innate across all social relationships.

Morality may be taught and developed even though it is innate (Cowell and Decety, 2015). Social relationships create a suitable environment for individuals to share, discuss and negotiate their dilemmas with ease. Thus, social interactions equip individuals with ample space to shape their moral skills (Lapsley et al., 2022). People exposed to a wide range of constructive social interactions can better negotiate their problems than individuals exposed to less constructive interactions. According to Mammen et al. (2019), the interaction between a child and a coequal peer is more productive than an interaction between a child and a parent. A child discusses their moral problems more with a peer than with a parent. Based on this, moral development is enabled by child–peer interactions more than child–parent interactions.

Moral development is not fostered by social interactions only; it can also be encouraged by exposing minds to various constructive and interactive environmental factors (Malle, 2021). Children’s learning environments are seen to promote moral development the best. Quality learning environments set the stage for exploration and socio-emotional growth. When children are presented with a warm, welcoming, culturally motivating and familiar environment, they feel comfortable and safe. The attractive spaces that adults prepare for children communicate expectations of responsibility and cooperative union (Hamlin, 2013a).

A well-designed environmental space with ample resources is considered fit for moral development. In such spaces, children can freely utilise the resources available to make judgements based on their interests (Paris et al., 2021). These environments have various characteristics: challenging and developmentally appropriate materials, ample supply of materials, appropriately sized small-group activities, a variety of small-group activities within a range of adult supervision, aesthetically appealing spaces to be with others and spaces to be alone, furnishings and materials accessible to children, displays of children’s work and support for children’s active engagement.

Additionally, the ample space provided by such an environment allows the concerned individuals to interact freely. The interaction between a child and an adult also gives rise to good opportunities for resolving moral dilemmas; this is done by posing questions and discussing them.

The three areas in which the selected studies have been categorized are: “Natural Moral Sense,” “Social Relationships and Moral Development,” and “Environmental Factors and Moral Development.” Although each approach proposes a different aetiology of the moral sense, the review brings out an agreement among the authors regarding the presence of a predisposition to morally acceptable behaviours (Cowell and Decety, 2015; Dahl, 2019; Korotaeva and Chugaeva, 2019) that mature and are influenced by life experiences determined by interpersonal relationships and environmental factors.

In general, this systematic review summarises the findings from various research studies and articles and provides a definite conclusion. First, moral sense is innate. Second, moral development is considerably strengthened through social interactions and exposure to constructive and interactive environmental factors.

### Limitations

The number of studies included in this systematic review was small. This can be mainly attributed to the strict criteria used during the inclusion and exclusion process. To establish a consensus on a specific area of study, numerous articles need to be included. Therefore, the small number of the studies analysed in this systematic review limits the accuracy of the conclusion reached. Limited data available for a systematic review is considered to limit the consensus achieved.

Furthermore, only items reported in English and those meeting the required study characteristics were considered relevant for this systematic review. Items that were reported in languages other than English, irrespective of their usefulness, were declared irrelevant for this systematic review. The use of articles published in a single language is a source of bias. Research studies reported in other languages are never promoted.

## Conclusion

The emergence and development of moral sense is a complex theme that has attracted the attention of multiple researchers. Several of these have focused their efforts on obtaining explicit knowledge of moral sense, moral development and the source of morality. Indeed, research studies vary in terms of the conclusions drawn. Therefore, this systematic review collated the various conclusions to reach a reasonable consensus, that the moral sense is a natural ability that every human is born with, and this natural ability can be nurtured through social interactions and by environmental factors.

The findings of the present review are confirmed by Wynn et al. (2018) who affirm that, although there is evidence of an early emerging moral sense, altruistic behaviours are selective from the first moments after birth. Children recognise and prefer cases of fairness and kindness and prefer people who have been kind to them and family members over strangers. They also tend to make errors of judgement based on the belonging of the other to their own group or to an external group. Morality, therefore, matures during growth and is refined: adult morality is the result of an arduous process of development that involves exposure to culture and the exercise of rationality.

An individual’s interaction with others, especially coequal people, allows moral problems to be shared, discussed and negotiated. This interactive behaviour allows the people in a conversation to explore new skills and knowledge that can significantly boost their moral judgements. Through social interactions, moral development is guaranteed. Moreover, environmental factors contribute to the development of moral capabilities too. As stated earlier, humans possess innate moral abilities. Thus, exposure of the prepared minds to the environmental aspects further shapes human morality. This systematic review mainly focused on children, and it was found that exposing children to environmental factors such as ample access to educational items, enables them to fully experience their interests and abilities. Therefore, moral sense is found to be naturally occurring; it can further be developed through exposure to constructive and interactive environments and social relationships. In conclusion, the questions of this systematic review have been answered.

## Data Availability Statement

The raw data supporting the conclusions of this article will be made available by the authors, without undue reservation.

## Author Contributions

PL wrote introduction and conclusion. Both authors contributed to the article and approved the submitted version.

## Conflict of Interest

The authors declare that the research was conducted in the absence of any commercial or financial relationships that could be construed as a potential conflict of interest.

## Publisher’s Note

All claims expressed in this article are solely those of the authors and do not necessarily represent those of their affiliated organizations, or those of the publisher, the editors and the reviewers. Any product that may be evaluated in this article, or claim that may be made by its manufacturer, is not guaranteed or endorsed by the publisher.
